# Postoperative Nausea and Vomiting After Deep Sedation for Radiofrequency Catheter Ablation in Pediatric Patients

**DOI:** 10.1002/joa3.70415

**Published:** 2026-07-06

**Authors:** Yoshikatsu Takeda, Shuhei Fujita, Naoto Sakumura, Kazuyuki Ueno, Kengo Miyashita, Takeshi Futatani, Akio Chikata, Kazuo Usuda, Hidenori Iwasaki, Taichi Nakamura, Kunio Ohta, Taizo Wada

**Affiliations:** ^1^ Department of Pediatrics Toyama Prefectural Central Hospital Toyama Japan; ^2^ Department of Pediatrics, School of Medicine, Institute of Medical, Pharmaceutical and Health Sciences Kanazawa University Ishikawa Japan; ^3^ Department of Cardiology Toyama Prefectural Central Hospital Toyama Japan

**Keywords:** complication, deep sedation, pediatric, postoperative nausea and vomiting, radiofrequency catheter ablation

## Abstract

Radiofrequency catheter ablation (RFA) effectively treats pediatric arrhythmias, but postoperative nausea and vomiting (PONV) is a common complication impacting patient experience. This study investigated PONV incidence and its contributing factors in pediatric RFA under deep sedation. This retrospective, single‐center cohort study reviewed 70 RFA procedures in 63 pediatric patients (aged ≤ 18 years) performed between January 2013 and December 2025. All procedures were managed under a standardized deep sedation protocol. Data on demographics, arrhythmia type, procedural characteristics (including ablation site: right‐sided vs. left‐sided), anesthetic management, and the occurrence of PONV within 24 h were collected. PONV occurred in 12 of the 70 procedures (17%). The incidence of PONV was significantly higher following left‐sided ablation compared to right‐sided ablation (8/24 [33%] vs. 4/46 [9%]; *p* = 0.017). Notably, there were no significant differences in procedure time, anesthesia time, number of radiofrequency applications, or the types and dosages of anesthetic agents administered between patients who developed PONV and those who did not. In the majority of these patients, PONV symptoms resolved spontaneously within 24 h without the need for rescue medications. The incidence of PONV was 17% in pediatric patients during RFA under deep sedation. Our findings suggest that most pediatric PONV under deep sedation is transient and clinically manageable without additional medical intervention, although careful observation remains essential during the early postoperative period.

## Graphical abstract

This retrospective study investigated the incidence of postoperative nausea and vomiting (PONV) in 70 pediatric radiofrequency catheter ablation procedures under deep sedation. PONV occurred in 17% of procedures, with a significantly higher incidence following left‐sided ablation. Importantly, the majority of PONV symptoms resolved spontaneously within 24 h without requiring rescue antiemetics. These findings suggest that pediatric PONV under deep sedation is generally transient and clinically manageable.

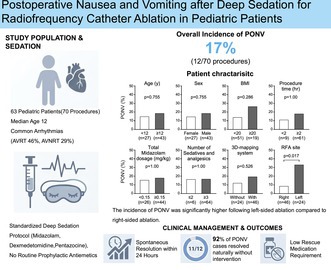

## 1 Introduction

Radiofrequency catheter ablation (RFA) has become an established and effective treatment for various cardiac arrhythmias [1]. It has demonstrated high efficacy in pediatric patients, with acute procedural success rates widely reported to exceed 90%, highlighting the significant clinical benefit of this therapy even in young cohorts [2]. Consequently, the number of patients where RFA is performed in pediatric arrhythmia management has been steadily increasing. However, pediatric patients often experience anxiety and pain, making it difficult to maintain the necessary stillness required for a successful procedure. Patient movement during RFA can compromise procedural success and potentially increase the risk of complications, requiring the use of sedation. Postoperative nausea and vomiting (PONV), a common complication following surgical and interventional procedures, causes pain and is a factor that reduces satisfaction as a perioperative complication. The incidence of PONV in children has been reported to be twice as high as that in adults [3]. This risk in pediatric patients underscores the need to develop strategies to minimize PONV in this vulnerable group. PONV can be particularly detrimental in the context of RFA in pediatric patients. Arrhythmias may recur after ablation, and a prior experience of PONV could result in trauma and subsequent aversion toward future procedures, making this complication a particular concern. Minimizing PONV is essential in optimizing the patient's experience and compliance with care. This study aimed to provide a better understanding of the prevalence of PONV within our specific treatment protocol and explore factors that may contribute to its occurrence.

## 2 Methods

### 2.1 Study Design and Population

This study was a retrospective, single‐center cohort study. We reviewed the medical records of all pediatric patients who underwent RFA for cardiac arrhythmias at Toyama Prefectural Central Hospital between January 1, 2013, and December 31, 2025. The inclusion criterion required that the patients were aged ≤ 18 years at the time of the ablation procedure. We excluded those whose medical records indicated that they did not receive any sedatives or analgesics during the procedure, as these patients are not representative of standard sedation practices. The study population was selected to capture patients who accurately represent the pediatric RFA cohort at our center.

### 2.2 Definition of PONV

We defined PONV as the occurrence of either vomiting or nausea within a 24‐h period after starting catheter ablation. Vomiting was defined as at least one episode of forceful expulsion of gastric contents. Nausea was defined as a subjective report of an unpleasant sensation in the stomach, frequently preceding vomiting, or an uncomfortable, sick, or nauseated feeling. We selected this definition to capture both objective and subjective signs of PONV. To objectively evaluate the clinical severity of PONV, we employed the concept of clinically significant PONV, which is well‐established in previous literature [4]. In this study, PONV was classified into two clinical categories based on objective parameters available in the medical records: “clinically significant” (defined as requiring rescue antiemetics, causing secondary complications, or lasting > 24 h) and “clinically insignificant” (resolving spontaneously without medication within 24 h).

### 2.3 Data Collection

We collected data from the electronic medical records of eligible patients, including patient demographics (age, sex, weight, height, body mass index [BMI], and BMI‐standard deviation score [SDS]), type of arrhythmia (e.g., atrioventricular reciprocating tachycardia [AVRT], atrioventricular nodal reentrant tachycardia [AVNRT], focal atrial tachycardia [FAT], atrial flutter [AFL], ventricular tachycardia [VT], and premature ventricular contractions [PVC]), specific procedural characteristics (e.g., ablation site, the use of a three‐dimensional [3D] mapping system, procedure duration, and number of RF applications), anesthetic management details (e.g., medications used for induction and maintenance of sedation, dosages, and duration of anesthesia), and the occurrence of PONV. The medication use data focused on the sedatives, analgesics, and antiemetics administered. We also recorded the type and timing of administration of all antiemetics administered.

### 2.4 Anesthetic Protocol and RFA Details

At our institution, pediatric patients undergoing RFA were managed with a standardized deep sedation protocol. Midazolam was used to administer anesthesia intravenously. Following induction, dexmedetomidine was administered intravenously to maintain a deep sedation level. To ensure adequate sedation, additional midazolam boluses were administered based on the clinical need. Pentazocine was administered as a preoperative analgesic with dosages based on the patient's weight. Although midazolam, pentazocine, and dexmedetomidine are typically used in combination for sedation in many patients, some patients may involve the use of only one or a subset of these agents. Furthermore, thiopental was occasionally used for sedation in certain instances. One dedicated doctor and two nurses were in charge of providing deep sedation. Regarding antiemetic strategies, prophylactic antiemetics were not routinely administered before or after the procedure. Instead, intravenous metoclopramide was utilized as a rescue medication only when clinically necessary, such as in patients with problematic vomiting.

All patients underwent electrophysiological studies (EPS) before RFA, and isoproterenol was used to induce arrhythmia. The procedural site was determined during EPS. Procedures involving the right atrium or right ventricle were defined as right‐sided ablation, and the left atrium and left ventricle were defined as left‐sided ablation. Some patients underwent RFA utilizing 3D mapping systems, which was decided at the discretion of the electrophysiologist based on the complexity of each patient. When using 3D mapping, the Rhythmia electroanatomic mapping system (Boston Scientific, Cambridge, MA, USA) was used for most patients. The EnSite NavX System (Abbott, St. Paul, MN, USA) was used for mapping in patients with verapamil‐sensitive VT. For left‐sided ablation, all patients had a transseptal puncture utilizing the Brockenbrough technique. Heparin was administered intravenously upon arterial and venous access, and the activated clotting time was maintained at > 300 s. All procedures were performed with patients spontaneously breathing and rarely requiring airway manipulation. Following the completion of the ablation procedure, hemostasis at the femoral puncture sites was primarily achieved by manual compression, followed by the application of a compressive dressing. Hemostatic devices were additionally utilized in certain patients at the operator's discretion. In accordance with our standardized post‐ablation management protocol, all patients were required to maintain strict bed rest in the supine position for 6 h after returning to the ward. The compressive dressing was maintained until the following morning.

### 2.5 Anatomical Classification of Ablation Sites

In this study, the anatomical locations of the ablation sites were classified using a modified clock‐face model along the tricuspid and mitral annuli, simulated in a left anterior oblique projection. The regions were categorized into three main domains: right‐sided, left‐sided, and the shared posteroseptal (PS) region. For the right‐sided (tricuspid valve) annulus, the regions were defined as: right anterior (RA, 10 to 1 o'clock), right posterior (RP, 5 to 7 o'clock), and right lateral (RL, 7 to 10 o'clock). For the left‐sided (mitral valve) annulus, the regions were defined as: left anterolateral (LAL, 12 to 2 o'clock), left lateral (LL, 2 to 5 o'clock), and left posterior (LP, 5 to 7 o'clock). The PS was defined as an independent, shared anatomical area bridging the tricuspid valve (4 to 5 o'clock) and the mitral valve (7 to 8 o'clock), encompassing the coronary sinus ostium and its proximal portion.

### 2.6 Statistical Analysis

Statistical analysis was performed using EZR (Saitama Medical Center, Jichi Medical University, Saitama, Japan), a graphical user interface for R (The R Foundation for Statistical Computing, Vienna, Austria). Continuous data were presented as median (IQR), and categorical data were presented as percentages. The Mann–Whitney *U* test was used to compare continuous variables. Fisher's exact test was used to analyze categorical data. A *p*‐value of < 0.05 was considered statistically significant.

## 3 Results

### 3.1 Study Flow

During the study period, 70 RFA procedures were performed on 63 patients. Of these, 12 (17%) developed PONV, and 58 procedures (83%) did not experience PONV.

### 3.2 Patient Demographics

Table 1 summarizes the demographic and procedural characteristics of the 70 procedures (in 63 individual patients) included in the study. The median age of the cohort was 12 years (IQR, 11–14), and the group comprised 27 female patients (39%). The median BMI was 18.5 kg/m^2^ (IQR, 16.6–20.5), and the median BMI‐SDS was 0.11 (IQR, −0.46 to 0.69). The median procedure time was 180 min (IQR, 150–216), the median anesthesia time was 193 min (IQR, 172–240), and the median number of RF applications was 9 (IQR, 4–19). The most prevalent arrhythmia was AVRT, observed in 32 procedures (46%). Other arrhythmias included AVNRT in 20 procedures (29%), AFL in 7 procedures (10%), VT in 7 procedures (10%), FAT in 5 procedures (7%), and PVC in 2 procedures (3%). Overall, PONV occurred in 12 procedures (17%).

**TABLE 1:** Characteristics of the procedures.

All procedures (*n* = 70)	
Individual patients	63
Female, *n* (%)	27 (39%)
Age (year), median (IQR)	12 (11–14)
Body weight (kg), median (IQR)	41.7 (33.8–54.2)
Height (cm), median (IQR)	151 (139–163)
BMI (kg/m^2^), median (IQR)	18.5 (16.6–20.5)
BMI‐SDS, median (IQR)	0.11 (−0.46 to 0.69)
PONV, *n* (%)	12 (17%)
Procedure time (min), median (IQR)	180 (150–216)
Use of 3D‐mapping system, *n* (%)	46 (66%)
Left‐sided ablation, *n* (%)	24 (34%)
Number of RF applications, median (IQR)	9 (4–19)
Isoproterenol use, *n* (%)	60 (86%)
Anesthetic management and drugs	
Anesthesia time (min), median (IQR)	193 (172–240)
Pentazocine use, *n* (%)	68 (97%)
Pentazocine (mg/kg), median (IQR)	0.28 (0.22–0.41)
Midazolam use, *n* (%)	69 (99%)
Midazolam (mg/kg), median (IQR)	0.18 (0.13–0.22)
Thiopental use, *n* (%)	15 (21%)
Thiopental (mg/kg), median (IQR)	3.8 (1.3–4.8)
Dexmedetomidine use, *n* (%)	61 (87%)
Betamethasone use, *n* (%)	23 (33%)
Type of arrhythmia, *n* (%)	
AVRT	32 (46%)
AVNRT	20 (29%)
AFL	7 (10%)
VT	7 (10%)
FAT	5 (7%)
PVC	2 (3%)

Abbreviations: 3D, three‐dimensional; AFL, atrial flutter; AVNRT, atrioventricular nodal reentrant tachycardia; AVRT, atrioventricular reciprocating tachycardia; BMI, body mass index; BMI‐SDS, body mass index standard deviation score; FAT, focal atrial tachycardia; IQR, interquartile range; PONV, postoperative nausea and vomiting; PVC, premature ventricular contractions; RF, radiofrequency; VT, ventricular tachycardia.

**FIGURE 1:**
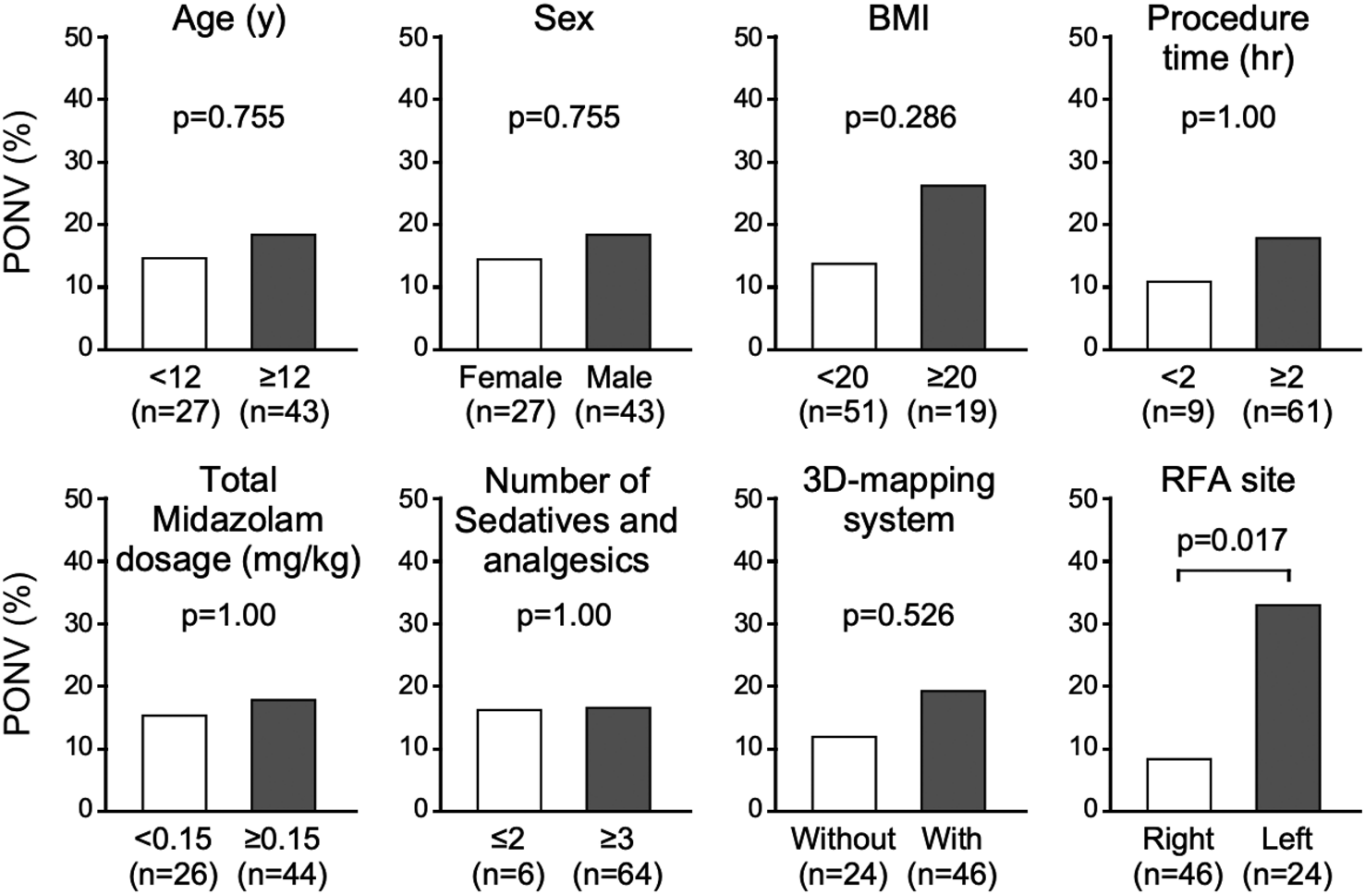
Incidence of postoperative nausea and vomiting (PONV) according to patient demographics and procedural characteristics. The bar graphs demonstrate the incidence of PONV stratified by age, sex, body mass index (BMI), procedure time, total midazolam dosage, number of sedatives and analgesics, use of a 3D‐mapping system, and radiofrequency ablation (RFA) site. There were no statistically significant differences in the incidence of PONV across most baseline and procedural variables. However, the incidence of PONV was significantly higher following left‐sided ablation compared to right‐sided ablation (*p* = 0.017). BMI, body mass index; PONV, postoperative nausea and vomiting; RFA, radiofrequency catheter ablation.

There were no statistically significant differences in age, sex, BMI, BMI‐SDS, height, body weight, procedure time, anesthesia time, number of RF applications, or the number of sedatives and analgesics administered when comparing patients who developed PONV with those who did not. Left‐sided ablation was performed in 24 procedures (34%) overall. However, the site of ablation was significantly associated with the incidence of PONV. Specifically, left‐sided ablation was significantly more common among procedures in the PONV group compared to the non‐PONV group (8/12 [67%] vs. 16/58 [28%]; *p* = 0.017) (Figure 1). Consequently, among patients who experienced PONV, 67% had undergone left‐sided ablation, while 33% had undergone right‐sided ablation. Furthermore, it is noteworthy that none of the procedures for AVNRT were associated with PONV (0/20 [0%]), and this absence of PONV was statistically significant (*p* = 0.015).

### 3.3 A Comparison of Right‐Sided and Left‐Sided Ablation

Table 2 presents the characteristics of the procedures, comparing right‐sided and left‐sided ablation. A total of 70 procedures were included in this analysis, with 24 undergoing left‐sided ablation and 46 undergoing right‐sided ablation. There were no significant differences between left‐sided and right‐sided ablation procedures in median procedure time, use of a 3D‐mapping system, use of isoproterenol, or median anesthesia time. However, the median number of RF applications was significantly higher in the right‐sided group compared to the left‐sided group (13 [IQR, 6–22] vs. 6 [IQR, 3–12]; *p* = 0.011). Regarding anesthetic management, there were no significant differences between left‐sided and right‐sided ablation in the number of procedures involving pentazocine administration or its median dosage, the number of procedures involving midazolam administration or its median dosage, the number of procedures involving thiopental administration or its median dosage, the use of dexmedetomidine, or the use of betamethasone. The incidence of PONV was significantly higher following left‐sided ablation procedures compared to right‐sided ablation procedures (8/24 [33%] vs. 4/46 [9%]; *p* = 0.017). This corresponded to an absolute risk difference of 24.6% (95% confidence interval [CI]: 4.1%–45.2%), a risk ratio of 3.83 (95% CI: 1.28–11.45), and an odds ratio of 5.11 (95% CI: 1.18–26.53). Furthermore, to address potential confounding between the ablation site and the arrhythmia type, we performed a multivariable logistic regression analysis including the ablation site and the presence of AVRT. In this adjusted model, left‐sided ablation demonstrated a strong trend toward being an independent predictor of PONV, although it did not reach statistical significance (odds ratio, 4.47; 95% CI: 0.98–20.4; *p* = 0.053). The distribution of primary arrhythmias showed several significant differences. AVRT was significantly more prevalent in the left‐sided ablation group compared to the right‐sided group (19/24 [79%] vs. 13/46 [28%]; *p* < 0.01). Conversely, AVNRT was significantly more common in right‐sided ablation procedures than in left‐sided procedures (19/46 [41%] vs. 1/24 [4%]; *p* < 0.01). There were no significant differences in the occurrence of AFL, VT, FAT, or PVC between left‐sided and right‐sided ablation procedures.

**TABLE 2:** Characteristics of procedures: A comparison of right‐sided and left‐sided ablation.

	Right‐sided ablation (*n* = 46)	Left‐sided ablation (*n* = 24)	*p*
Procedure time (min), median (IQR)	182 (154–207)	175 (150–240)	0.781
Use of 3D‐mapping system, *n* (%)	28 (61%)	18 (75%)	0.295
Number of RF applications, median (IQR)	13 (6–22)	6 (3–12)	0.011*
Isoproterenol, *n* (%)	40 (87%)	20 (83%)	0.727
Anesthesia time (min), median (IQR)	193 (173–239)	197 (162–242)	0.97
Pentazocine use, *n* (%)	44 (96%)	24 (100%)	0.543
Pentazocine (mg/kg), median (IQR)	0.30 (0.24–0.44)	0.24 (0.19–0.31)	0.084
Midazolam use, *n* (%)	45 (98%)	24 (100%)	1
Midazolam (mg/kg), median (IQR)	0.18 (0.13–0.24)	0.16 (0.13–0.20)	0.371
Thiopental use, *n* (%)	11 (24%)	4 (17%)	0.554
Thiopental (mg/kg), median (IQR)	4.4 (2.5–5.1)	1.9 (1.2–3.0)	0.343
Dexmedetomidine use, *n* (%)	38 (83%)	23 (96%)	0.151
Betamethasone use, *n* (%)	18 (39%)	5 (21%)	0.18
Type of arrhythmia, *n* (%)			
AVRT	13 (28%)	19 (79%)	< 0.01*
AVNRT	19 (41%)	1 (4%)	< 0.01*
AFL	7 (15%)	0 (0%)	0.087
VT	3 (7%)	4 (17%)	0.221
FAT	4 (9%)	1 (4%)	0.654
PVC	1 (2%)	1 (4%)	1

*Note:* Asterisks indicate statistically significant differences.

Abbreviations: 3D, three‐dimensional; AFL, atrial flutter; AVNRT, atrioventricular nodal reentrant tachycardia; AVRT, atrioventricular reciprocating tachycardia; FAT, focal atrial tachycardia; PONV, postoperative nausea and vomiting; PVC, premature ventricular contractions; VT, ventricular tachycardia.

### 3.4 Detailed Review of the PONV Patients

**FIGURE 2:**
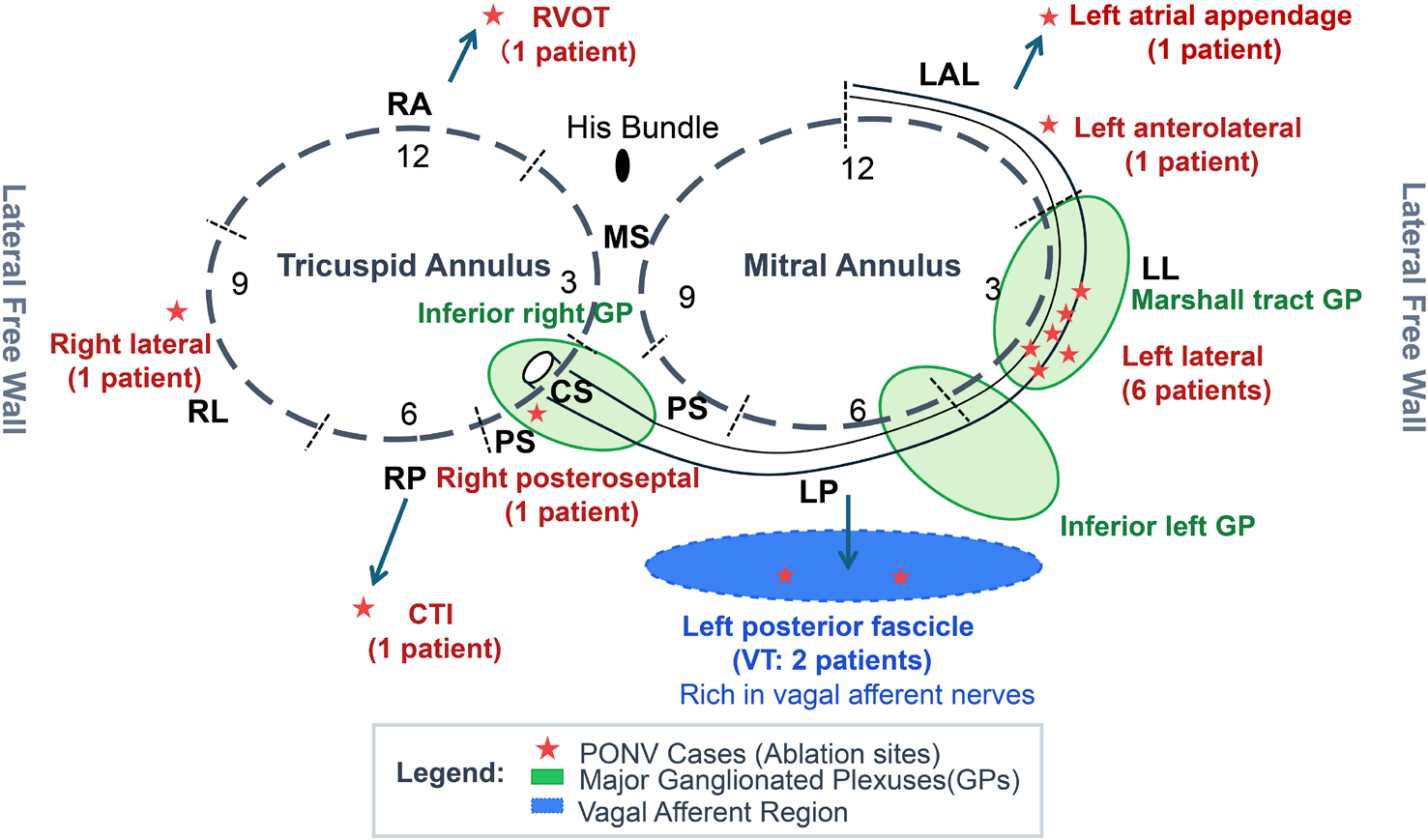
Anatomical distribution of all 12 PONV patients in relation to emetogenic autonomic pathways. This schematic diagram illustrates the location of radiofrequency ablation sites for all patients who experienced PONV, separated into right‐sided and left‐sided procedures. The green shaded areas represent the major intrinsic cardiac ganglionated plexuses (GPs), and the red stars (★) indicate the approximate ablation sites associated with PONV. Right‐sided, 4 patients: Shows a scattered distribution. While one AVRT patient occurred at the right posteroseptal region (in close proximity to the inferior right GP), the remaining patients occurred in anatomical regions generally devoid of major GP clusters, such as the right lateral wall, cavotricuspid isthmus (CTI), and right ventricular outflow tract (RVOT). Left‐sided, 8 patients: Demonstrates a striking anatomical concordance. Ablation sites for left‐sided AVRT (left lateral and anterolateral regions) heavily overlap with the superior left and inferior left GPs. Additionally, the target for the 2 VT patients (left posterior fascicle) is a posteroinferior ventricular region known for its rich distribution of vagal afferent nerves. AVRT, atrioventricular reciprocating tachycardia; CTI, cavotricuspid isthmus; GP, ganglionated plexus; LAL, left anterolateral; LL, left lateral; LP, left posterior; MS, mid‐septal; PONV, postoperative nausea and vomiting; PS, posteroseptal; RA, right anterior; RL, right lateral; RP, right posterior; RVOT, right ventricular outflow tract; VT, ventricular tachycardia.

Table 3 presents the detailed data of the 12 patients who experienced PONV, which included four female and eight male patients. Most patients had an underlying arrhythmia of AVRT (eight patients). Among the remaining patients, two patients had VT, one patient had FAT, and one patient had PVC. Left‐sided ablation was performed in the majority of patients with PONV (8 out of 12 patients). Nine of the 12 patients underwent ablation using a 3D‐mapping system. The median time from hemostasis to the onset of PONV was 2.5 h (interquartile range, 1.8–5.7 h). Based on our severity grading, 10 of the 12 PONV patients (83%) were classified as clinically insignificant, resolving spontaneously without rescue antiemetics within a few hours. Only 2 patients (17%) were classified as clinically significant: a rescue antiemetic (intravenous metoclopramide) was administered in only one of the 12 patients due to puncture site bleeding caused by nausea‐induced body movements, while the remaining 11 patients recovered without any antiemetic medications. Furthermore, none of the patients in our cohort reported psychological trauma or expressed an aversion to future procedures due to their PONV experience. In the single patient whose PONV persisted for more than 24 h, a head magnetic resonance imaging (MRI) scan confirmed the absence of cerebral infarction or hemorrhage. Overall, no severe complications were observed in our cohort. Figure 2 illustrates the anatomical distribution of the ablation sites for all 12 PONV patients. Notably, the majority of the ablation sites in these patients (9 out of 12 patients, 75%) were located in close anatomical proximity to highly emetogenic autonomic structures. Specifically, 7 patients were situated near the major intrinsic cardiac ganglionated plexuses (GPs), and 2 patients were located in a region rich in vagal afferent nerves.

**TABLE 3:** Characteristics of patients with PONV.

No.	Age (year)	Sex	Type of arrhythmia	RFA site	Type of 3D mapping	Midazolam (mg/kg)	Pentazocine (mg/kg)	Thiopental (mg/kg)	Dexmede‐ tomidine	Betame‐thasone	Number of RF application	Procedure time (min)	Anesthesia time (min)
1	7	F	AVRT	Left lateral	Rhythmia	0.15	0.31	−	+	−	8	257	270
2	11	M	AVRT	Left lateral	Rhythmia	0.3	0.27	−	+	−	6	152	249
3	12	M	AVRT	Left lateral	−	0.15	0.31	−	+	+	1	118	160
4	12	F	AVRT	Right lateral	−	0.26	0.45	−	+	+	32	159	173
5	14	M	AVRT	Left lateral	Rhythmia	0.13	0.26	−	+	−	3	158	148
6	14	M	AVRT	Right posteroseptal	CARTO	0.16	0.26	0.9	+	−	7	207	190
7	16	M	AVRT	Left lateral	Rhythmia	0.13	0.17	−	−	−	5	122	108
8	5	F	AVRT, FAT	Left lateral, Left anterolateral, Left atrial appentage	Rhythmia	0.18	0.54	4.9	+	−	41	266	429
9	11	M	VT	Left posterior fascicle	EnSite	0.19	0.17	1.4	+	−	6	252	265
10	14	M	VT	Left posterior fascicle	EnSite	0.14	0.11	−	+	−	22	241	217
11	14	M	AFL	CTI	Rhythmia	0.24	0.35	−	+	−	9	129	133
12	17	F	PVC	RVOT	−	0.17	0.28	5.6	−	+	17	179	206

Abbreviations: 3D, three‐dimensional; AFL, atrial flutter; AT, atrial tachycardia; AVRT, atrioventricular reciprocating tachycardia; AVNRT, atrioventricular nodal reentrant tachycardia; CTI, cavotricuspid isthmus; PONV, postoperative nausea and vomiting; PVC, premature ventricular contractions; RF, radiofrequency; RVOT, right ventricular outflow tract; VT, ventricular tachycardia.

## 4 Discussion

This study provides valuable insights into PONV following RFA in pediatric patients undergoing deep sedation. Our analysis showed that the overall incidence of PONV following pediatric RFA was lower than that previously reported. Moreover, left‐sided ablation procedures were significantly associated with a higher incidence of PONV, indicating a potential relationship between the ablation site and PONV development.

In this study, most patients were > 6 years old (median age: 12 years), which was consistent with the age range associated with a higher PONV risk. Pediatric patients are more likely to experience PONV than adults, with reported incidence rates of 5%, 20%, 35%, and 33% for those aged < 1 year, 1–5 years, 6–10 years, and 11–16 years, respectively [3]. Despite including many patients within this higher‐risk age range, the overall incidence of PONV in our study population (17%) was lower than previously reported rates of RFA in pediatric patients [5, 6, 7]. In previous reports, general anesthesia was performed more commonly during RFA procedures; however, in this study, patients were managed under deep sedation, and it appeared that the frequency of PONV incidence may be lower with deep sedation. The choice of sedation regimen significantly impacts PONV occurrence, as Matsui et al. reported [8]. While Sakanoue et al. demonstrated that the use of propofol under deep sedation reduced the PONV incidence to 5.5% [9], its routine introduction in our pediatric cohort is difficult due to potential side effects in children and our limited experience with its use in this setting. Importantly, our study demonstrated a PONV incidence of 17% even without propofol. We consider this rate to be not excessively high, suggesting that our current deep sedation protocol is clinically manageable.

Although there were no differences in other evaluation factors, PONV was more frequent in left‐sided ablation than in right‐sided ablation. One plausible, albeit hypothesis‐generating, pathophysiological mechanism is that stimulation of the cardiac autonomic nervous system during left‐sided procedures may induce bradycardia and/or hypotension, which are recognized emetogenic stimuli. This hypothesis is supported by the anatomical distribution of intrinsic cardiac ganglionated plexuses (GPs). Major GPs are predominantly located within atrial tissue, with a notably sparse distribution in the ventricles. Moreover, GPs are well‐established to be more densely concentrated in the left atrium than in the right [10]. Indeed, some left‐sided ablation sites in our study corresponded with known GP locations. Thus, RF applications near these GPs may have inadvertently stimulated the autonomic nervous system, contributing to the higher PONV incidence with left‐sided ablation.

Furthermore, our analysis revealed that the median number of RF applications was significantly higher in the right‐sided group compared to the left‐sided group. Generally, an increased number of RF applications correlates with longer procedure and anesthesia times, as well as a higher total dose of anesthetic agents, which are traditional risk factors for PONV. However, in our cohort, there were no significant differences in procedure time, anesthesia time, or the overall number of RF applications between the PONV and non‐PONV groups. This finding suggests that the specific anatomical site of ablation—namely, proximity to the highly emetogenic GPs in the left heart—may be an important contributing factor to PONV risk, potentially independent of the mere frequency of RF applications or the associated cumulative anesthetic exposure.

Another theoretical consideration for PONV following left‐sided procedures is the potential occurrence of micro‐cerebral thromboembolic events. Some studies in adults have suggested that silent cerebral infarctions can occur after RFA [11, 12, 13, 14, 15]. In our cohort, one patient experienced prolonged PONV persisting for more than 24 h, accompanied by a headache. A subsequent head MRI scan was performed and successfully ruled out cerebral infarction and hemorrhage. This patient highlights that a subclinical embolic event could theoretically present with isolated nausea, emesis, or headache. Notably, we do not advocate for routine cerebral imaging after left‐sided ablation, as almost all patients in our cohort recovered from PONV within 24 h without neurological sequelae. However, based on our clinical experience and from a safety perspective, brain imaging should be strongly considered to rule out neurological complications if PONV is unusually severe, accompanied by neurological symptoms such as headache, or persists for more than 24 h.

Finally, our comparative analysis of left‐ versus right‐sided ablation procedures revealed a clear distinction dictated by the arrhythmia type. AVRT procedures were predominantly left‐sided, while AVNRT procedures were mainly right‐sided. This pattern in AVRT aligns with existing literature on pediatric RFA, where left‐sided accessory pathways (APs) are reported to have a higher prevalence than right‐sided or septal APs [2]. This reported prevalence supports our observation of a higher incidence of left‐sided RFA for AVRT. In AVNRT, slow pathway ablation is performed, which is why a right‐sided approach is utilized in most patients.

## 5 Limitations

Our study has several limitations. First, as a retrospective, single‐center study with a relatively small sample size, the statistical power of our analysis was limited, and a randomized control group was lacking. Second, the retrospective design precluded the use of standardized pediatric PONV scoring systems. Our reliance on binary (yes/no) chart documentation to define PONV may have introduced a measurement bias. Third, the precise mechanisms underlying the higher incidence of PONV in left‐sided procedures—including the potential involvement of cerebral microembolism or autonomic stimulation—remain hypotheticals that could not be proven with our data. Therefore, a prospective, multicenter study with a larger sample size is needed to validate our findings and further assess these underlying mechanisms.

## 6 Conclusion

The incidence of PONV was 17% in pediatric patients during RFA under deep sedation. Our findings suggest that the majority of patients resolved spontaneously within 24 h without additional medical intervention. Although careful observation is necessary in the early postoperative phase, our study indicates that most pediatric PONV under deep sedation is clinically manageable and follows a favorable clinical course.

## Ethics Statement

Approval of the research protocol: Approval was granted by the Institutional Review Board of Toyama Prefectural Central Hospital (Reference No. 67‐17).

## Consent

Written informed consent was obtained from the parents of all patients.

## Conflicts of Interest

The authors declare no conflicts of interest.

## Data Availability

Research data are not shared.
